# Resilience in Adult Coeliac Patients on a Gluten-Free Diet: A Cross-Sectional Multicentre Italian Study

**DOI:** 10.3390/nu16162595

**Published:** 2024-08-07

**Authors:** Annalisa Schiepatti, Stiliano Maimaris, Simona Randazzo, Daria Maniero, Roland Biti, Giacomo Caio, Lisa Lungaro, Antonio Carroccio, Aurelio Seidita, Davide Scalvini, Carolina Ciacci, Federico Biagi, Fabiana Zingone

**Affiliations:** 1 Department of Internal Medicine and Therapeutics, University of Pavia, Italy, 27100 Pavia, Italy; annalisa.schiepatti01@universitadipavia.it (A.S.); stiliano.maimaris01@universitadipavia.it (S.M.); simona.randazzo01@universitadipavia.it (S.R.); davide.scalvini01@universitadipavia.it (D.S.); federico.biagi@icsmaugeri.it (F.B.); 2 Gastroenterology Unit of Pavia Institute, Istituti Clinici Scientifici Maugeri IRCCS, 27100 Pavia, Italy; 3 Department of Surgery, Oncology and Gastroenterology, University of Padova, 35128 Padua, Italy; dariamaniero@gmail.com (D.M.); roland.biti@studenti.unipd.it (R.B.); 4 Department of Translational Medicine, St. Anna Hospital, University of Ferrara, 44121 Ferrara, Italy; caigmp@unife.it (G.C.); lisa.lungaro@gmail.com (L.L.); 5 Mucosal Immunology and Biology Research Center, Massachusetts General Hospital-Harvard Medical School, Boston, MA 02114, USA; 6 Unit of Internal Medicine, “V. Cervello” Hospital, Ospedali Riuniti “Villa Sofia-Cervello”, 90146 Palermo, Italy; antonio.carroccio@unipa.it (A.C.); aurelio.seidita@unipa.it (A.S.); 7 Department of Health Promotion Sciences, Maternal and Infant Care, Internal Medicine and Medical Specialties (PROMISE), University of Palermo, 90127 Palermo, Italy; 8 Institute for Biomedical Research and Innovation (IRIB), National Research Council (CNR), 90146 Palermo, Italy; 9 Department of Medicine, Surgery and Dentistry, Scuola Medica Salernitana, University of Salerno, 84081 Salerno, Italy; cciacci@unisa.it; 10 Unit of Gastroenterology, Azienda Ospedale Università Padova, 35128 Padova, Italy

**Keywords:** coeliac disease, resilience, quality of life, gluten-free diet, burden, chronic disease

## Abstract

Background. Data on resilience, the ability to recover from adversity, in coeliac disease (CeD) are lacking. Aim. To assess the degree of resilience in patients with CeD on a gluten-free diet (GFD), and its association with clinical features, sociodemographic factors, psychological morbidity, and quality of life (QOL). Methods. A cross-sectional multicentre Italian study was conducted on adult CeD patients between May 2022 and April 2023. Connor–Davidson Resilience Scale (CD-RISC), the Coeliac Disease-specific Quality of Life Scale (CD-QOL), the State–Trait Anxiety Inventory scale (STAI-Y), and the Beck Depression Inventory scale (BDI) were used to evaluate resilience, QOL, anxiety, and depression, respectively. A multivariate analysis was conducted to identify factors independently associated with the degree of resilience. Results. A total of 305 patients (221 F, mean age at CeD diagnosis 36 ± 16 years) on a long-term GFD (median 8 years, IQR 3–17) were enrolled. A total of 298/305 patients (98%) had a high level of resilience (CD-RISC ≥ 35). At univariate analysis, resilience was statistically associated with male gender (*p* = 0.03), age at enrolment (*p* = 0.02), marital status (*p* = 0.03), QOL (*p* < 0.001), anxiety (*p* < 0.001), and depression (*p* < 0.001). On multivariate regression analysis, trait anxiety (STAI-Y2, *p* < 0.001) and depression (BDI, *p* = 0.02) were independent predictors of lower levels of resilience. Conclusions. Higher trait anxiety predicts lower levels of resilience. Targeted interventions in this subgroup of patients may be helpful for their management and follow-up.

## 1. Introduction

Coeliac disease (CeD) is a chronic immune-mediated enteropathy developing in genetically susceptible individuals after the ingestion of gluten and characterised by a prevalence of around 1% in the general population and a very heterogeneous clinical picture [1,2,3,4,5]. A certain degree of villous atrophy (VA) and positive IgA tissue transglutaminase/endomysial antibodies are the culprit for the diagnosis [1,2,3,4,5]. A gluten-free diet (GFD) is the cornerstone of treatment leading to resolution of clinical symptoms and small bowel histological lesions in the vast majority of patients [1,2,3,4,5]. Despite this, reaching and maintaining a strict dietary adherence is socially, economically, and psychologically demanding for many patients, particularly over the long-term [6,7,8]. Strategies to implement and maintain adequate dietary adherence and contrast poor long-term outcomes in CeD have been the focus in recent years [6,7,8]. Moreover, great attention has been dedicated also to the impact that the disease status itself and its treatment can have on coeliac patients’ global health and the subjective perception of the disease status by patients themselves. In this regard, psychological morbidity in CeD has emerged as a relevant problem, manifesting with anxiety, depression, fatigue, and mood disorders, either due to the disease course itself, or to the effort required to maintain strict adherence to a GFD [9,10,11,12,13,14]. As a consequence of this psychological burden, quality of life (QOL) in coeliac patients is often reduced and strategies to support patients are key requirements for delivering the best quality of care.

Resilience, which is defined as the ability of an individual to cope with and recover from adversity, is a psychological construct that can be modifiable with appropriate therapies [15,16,17]. This has become an area of interest for patients affected by chronic stress-sensitive disorders such as fibromyalgia and post-traumatic stress disorders [16,17,18,19], and more recently, also some gastrointestinal disorders such as irritable bowel syndrome (IBS) and inflammatory bowel disease (IBD) [20,21,22]. In patients affected by such chronic disorders, it has been shown that high levels of resilience were associated with lower levels of anxiety and better QOL [20,21,22].

Currently, data on resilience in coeliac patients and its relationship with psychological morbidity and QOL are lacking. There is only one US study, which primarily investigated resilience in patients affected by IBS, that also evaluated resilience in 17 coeliac patients as a control group. This study showed that IBS patients had similar resilience as those with other chronic GI conditions who presented with similar symptoms [20].

Therefore, with the present study we aimed to evaluate the relationship between resilience, anxiety, depression, QOL, and degree of adherence to a GFD in a multicentre Italian cohort of adult coeliac patients on a long-term GFD.

## 2. Patients and Methods

### 2.1. Study Design and Setting

This is a cross-sectional Italian multicentre study, which aimed to assess the level of resilience in adult coeliac patients on a GFD and to evaluate the relationship between degree of resilience, clinical features, psychological morbidity, QOL, and adherence to a GFD.

Patients taking part in the study were prospectively enrolled between May 2022 and April 2023 at five major Italian centres with expertise in the diagnosis and management of CeD (Padova, Pavia, Salerno, Palermo, and Ferrara).

### 2.2. Study Population

The study population consisted of adult coeliac patients (age at diagnosis > 18 years) on a GFD for at least 12 months, who agreed to participate to a survey to evaluate resilience, QOL, and psychological status. Participation in the study was proposed to coeliac patients when they attended the outpatient clinic for a follow-up visit. Patients who agreed to participate answered an anonymous online questionnaire to complete in approximately 10–15 min.

Each patient received a diagnosis of CeD on the basis of positive endomysial/tissue transglutaminase antibodies and a certain degree of VA on duodenal biopsies while on a gluten-containing diet, in accordance with major international guidelines [2,3,4,5].

### 2.3. Development of the Survey Questionnaire

The survey consisted of five independent questionnaires investigating specific domains that have already been previously validated. These questionnaires, which will be described in detail below, included the Connor–Davidson Resilience Scale (CD-RISC) [16], the Coeliac Disease-specific Quality of Life Scale (CD-QOL) [23,24], the State–Trait Anxiety Inventory (STAI-Y) [25], the Beck Depression Inventory (BDI) [26], and the Pavia Score for evaluating GFD adherence [27].

The Connor–Davidson Resilience Scale (CD-RISC) is the most widely used tool to evaluate psychological resilience [16]. The Connor–Davidson Resilience scale (CD-RISC) comprises 25 items, each rated on a 5-point scale (0–4), for a total final score ranging from 0 to 100 and with higher scores reflecting a greater level of resilience. An overall CD-RISC score < 35 indicates low resilience, whereas a CD-RISC score ≥ 35 indicates high resilience.

The CD-QOL is a reliable and valid measure of CeD-related QOL and it is based on the analysis of patients’ perception of their life during treatment with a GFD, taking into account 20 items across four clinically relevant subscales (limitations, dysphoria, health concerns, and inadequate treatment) [23]. For the purpose of our study, we have used the Italian version of the CD-QOL questionnaire [24].

The form Y of the State–Trait Anxiety Inventory (STAI-Y) is a commonly used measure of trait and state anxiety [25] consisting of 40 self-report items on a 4-point Likert scale. This form Y version has 20 items for assessing state anxiety (STAI-Y 1) and 20 for trait anxiety (STAI-Y 2). Higher scores are positively correlated with higher levels of anxiety.

The Beck Depression Inventory (BDI) is a 21-item, self-report rating inventory that measures characteristic attitudes and symptoms of depression. Each item is rated on a scale ranging from 0 to 3. The higher the final cumulative score, the greater the depressive state [26].

Adherence to a GFD was evaluated using the Pavia questionnaire. This is a five-level score (0 to 4), which was previously developed and validated in Pavia, Italy [27] to assess GFD adherence. Patients scoring between 0 and 2 were considered not adherent to a GFD, while patients scoring 3 or 4 were considered adherent.

### 2.4. Data Collection

Clinical and sociodemographic data including age and symptoms at diagnosis of CeD [28], gender, age at study enrolment, time on a GFD, persistence of symptoms despite a GFD, marital status, and educational level were collected for each patient. These data together with the data deriving from the survey based on CD-QOL, CD-RISC, STAI-Y, BDI, and Pavia GFD adherence questionnaires were collected in an ad hoc Excel spreadsheet anonymously.

### 2.5. Statistics

STATA 11 software was used for statistical analyses (Stata Corp., College Station, TX, USA). Normality of data distribution was evaluated using the Shapiro–Wilk test. Continuous variables were reported as mean and standard deviation (SD) or median and interquartile range (IQR), for skewed variables, and differences between two or more groups were assessed with the Wilcoxon rank-sum test and Kruskal–Wallis test, respectively. Categorical variables were reported as total count and percentages and compared among groups with the chi-square test. The correlations between resilience and other psychological scores were assessed using Spearman’s correlation coefficient (rho). The relationship between resilience (CD-RISC), clinical, psychologic, and sociodemographic variables was evaluated by means of linear regression analysis. *p*-values < 0.05 were considered statistically significant. A multivariate regression analysis was conducted by taking into account the variables with *p* < 0.05 on univariate analysis. The multivariate analysis was conducted by using the backward elimination technique.

### 2.6. Ethics

The protocol for the multicentre study was approved by the Ethical Review Board of the coordinating centre of Padova (protocol number 4680/AO/19, approved on 11 April 2019, extension approved on 5 May 2022).

The study protocol conforms to the ethical guidelines of the 1975 Declaration of Helsinki (6th revision, 2008) as reflected in a prior approval by the institution’s human research committee. All the patients answering the questionnaire gave their written consent to participate to the study. All data were collected and analysed in anonymous form.

## 3. Results

In the period between May 2022 and April 2023, 305 patients (221 F, mean age at CeD diagnosis 36 ± 16 years; median age at enrolment 48, IQR 35–60) on a long-term GFD (mean 11 ± 10 years) completed the survey and were included into the study. The clinical and sociodemographic characteristics of the patients are shown in Table 1.

The vast majority of coeliac patients (298/305, 98%) had a high level of resilience (CD-RISC ≥ 35). QOL scores were good overall (mean 81 ± 13). Anxiety scores were suboptimal (STAI-Y1 median 39, IQR 32–50; STAI-Y2 median 40, IQR 33–49). Depression scores were low overall (BDI median 7, IQR 3–14). Data for clinical pattern at diagnosis of CeD, adherence to a GFD, and persistence of symptoms during follow-up despite a GFD were only available for 182/305 (60%) patients. More than half of the patients were diagnosed because of non-classical symptoms and the vast majority (89%) had good adherence to a GFD (Pavia GFD score ≥ 3), and only a minority of patients (27%) still reported persistent symptoms despite a GFD at time of survey.

### 3.1. Correlation between Clinical Features, Resilience, Quality of Life, Anxiety, and Depression

Table 2 shows the relationship between clinical and demographic features with resilience, anxiety, depression, and QOL.

### 3.2. Resilience (CD-RISC)

Factors related to resilience included gender, age at enrolment in the study, and marital status. Higher levels of resilience were found in males than in females (CD-RISC median 73 vs. 68, *p* = 0.03). Resilience also differed according to age group, with patients aged < 35 years having lower levels of resilience than those aged 35–55 years and ≥55 years (CD-RISC median 67, 71, and 70, respectively, *p* = 0.02). Those in a stable relationship also had higher levels of resilience (CD-RISC median 70 vs. 68, *p* = 0.03). No statistically significant relationship was found between levels of resilience and clinical pattern at diagnosis, disease duration, persistence of symptoms despite a GFD, and degree of adherence to a GFD.

### 3.3. Anxiety (STAI-Y 1 and STAI-Y 2)

A statistically significant relationship was found between gender and anxiety. More precisely, women were statistically more likely than males to have higher values of both state (median STAI-Y1 41 vs. 35, *p* < 0.001) and trait anxiety (median STAI-Y2 42 vs. 36, *p* < 0.001).

Age at enrolment, marital status, and persistence of symptoms despite a GFD were also associated with trait anxiety. Higher levels of anxiety were found in patients ≤35 years old compared to those aged 35–55 years old and ≥55 years old (median STAI-Y2 44, 39, and 40, respectively, *p* = 0.0143), in those who were not in a stable relationship (median STAI-Y2 43 vs. 39, *p* = 0.02), and in those who still reported persistent symptoms despite a GFD (median STAI-Y2 42 vs. 39, *p* = 0.03).

### 3.4. Depression (BDI)

Gender, clinical pattern at diagnosis, marital status, persistent symptoms despite a GFD, and adherence to a GFD were statistically related to depression. More precisely, higher levels of depression were found in females than males (median BDI 8 vs. 3, *p* < 0.001), in patients who were diagnosed with classical symptoms (median BDI 9 vs. 7 for non-classical vs. 3 for asymptomatic, *p* = 0.04), in those who were not in a stable relationship (median BDI 9 vs. 6, *p* < 0.01), in those who had persistence of symptoms despite a GFD (median BDI 8 vs. 7, *p* < 0.01), and in those with poor GFD adherence (median BDI 10 vs. 7 for good GFD adherence, *p* = 0.048).

### 3.5. Quality of Life (CD-QOL)

Persistence of symptoms despite a GFD was the only factor associated with reduced QOL (median CD-QOL 76 vs. 86, *p* < 0.001). No statistically significant relationships emerged between QOL and gender, clinical pattern at diagnosis, age at enrolment, disease duration, educational level, marital status, and GFD adherence.

### 3.6. Adherence to a Gluten-Free Diet

Adherence to a GFD was good in the vast majority of patients (89%). Higher levels of depression were found in patients with poor adherence to a GFD (median BDI 10 vs. 7, *p* = 0.048). Patients with strict GFD adherence were more likely to have higher levels of resilience (median CD-RISC 68 vs. 59, *p* = 0.07), lower levels of anxiety (median STAI-Y1 39 vs. 48, *p* = 0.11; median STAI-Y2 41 vs. 47, *p* = 0.0621), and better QOL (median CD-QOL 84 vs. 77, *p* = 0.19), although these differences were not statistically significant.

### 3.7. Correlation Analysis

**Figure 1:**
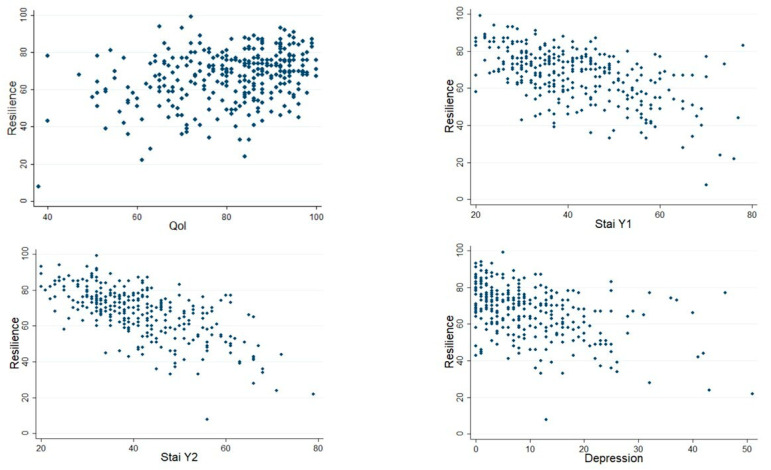
Correlation between levels of resilience, quality of life, anxiety, and depression.

Resilience score was weakly positively correlated with QOL score (rho = 0.27, *p* < 0.0001) but moderately negatively with STAI-Y1 (rho = −0.52, *p* < 0.001), STAI-Y2 (rho = −0.65, *p* < 0.001), and depression (rho = −0.45, *p* < 0.001). See scatter plots in Figure 1.

### 3.8. Linear Regression Analysis

Table 3 shows the results of regression analyses between the degree of resilience and the clinical, psychological, and socio-demographic factors considered into the study. On univariate analysis, the degree of resilience was directly correlated with age at enrolment (*p* = 0.01) and quality of life (CD-QOL, *p* < 0.001) and inversely correlated with gender (*p* = 0.02), marital status (*p* = 0.02), state anxiety (*p* < 0.001), trait anxiety (*p* < 0.001), and depression (*p* < 0.001).

However, at multivariate analysis only trait anxiety (inverse relationship, *p* < 0.001) and depression (direct relationship, *p* = 0.02) were independently related to resilience.

## 4. Discussion

This multicentre cross-sectional study, conducted in five major Italian referral centres for coeliac disease, has evaluated for the first time the relationship between resilience, psychological morbidity, and QOL in a large cohort of adult coeliac patients on a long-term GFD.

Resilience is a psychological individual innate construct defining the ability of a person to cope with stressful situations, and it can be modifiable by means of targeted interventions such as hypnosis and behavioural/cognitive interventions [15,16,17].

In the last years, the aspect of psychological morbidity has emerged as a strong determinant of QoL, thus becoming a prevalent area of intervention in many chronic gastrointestinal disorders, including CeD [9,10,11,12,13,14,29,30,31,32,33]. In particular, higher levels of resilience have been associated with better QOL, lower levels of anxiety, and better responses to therapies in patients affected by IBS and IBD [20,21,22,29,30,31,32,33,34]. Moreover, in other contexts, it has been shown that interventions that increased individual resilience were able to mitigate anxiety and depression [35,36,37].

Major findings of our study include the quantification of the levels of resilience in adult coeliac patients, which are overall good (CD-RISC > 35) in the vast majority of them (>90% of patients), and the identification of trait anxiety and depression as independent predictors of resilience. In our study cohort, patients showed good levels of resilience and QOL overall, with mild anxiety and depression levels overall. Although on a univariate analysis higher level of resilience directly correlated with better QOL and inversely correlated with anxiety and depression, on a multivariate analysis only trait anxiety and depression were identified as independently associated with resilience. However, while higher levels of trait anxiety were associated with lower resilience, low levels of depression were predictive of lower resilience.

The inverse relationship between trait anxiety and resilience can be explained by the bidirectional relationship between psychological morbidity and resilience, with it being true from one side that higher levels of resilience are associated with better individual strategies to cope with stressful situations and, from the other side, that lower levels of psychological morbidity led to higher resilience. Instead, the relationship between low levels of depression and low levels of resilience is certainly more difficult to explain, as we would have expected to find higher resilience in less depressed individuals.

Our results also show an association between persistence of symptoms despite a GFD and reduced QOL in coeliac patients. This confirms the necessity of developing targeted interventions for patients with persistent symptoms despite a GFD, which in our study affected nearly one third of patients, and up to 30–50% of all coeliac patients according to the literature [38,39,40]. Although it is well known that poor compliance with a GFD negatively affects QOL [12,41,42], and, therefore, resilience could potentially represent a target to implement GFD adherence and boost QOL, in our study no statistically significant relationship emerged between resilience, GFD adherence, and persistence of symptoms despite a GFD. Although the relationship between resilience and access to gluten-free foods was not directly investigated, there are two points that should be considered. First, in Italy, patients are provided with a national subsidy for purchasing gluten-free foodstuffs. This can significantly reduce the economic burden associated with following a gluten-free diet, unlike many other countries. Secondly, there is no doubt that the cultural and culinary background in Italy allow patients to have a wider choice of gluten-free foodstuffs to choose from.

A major strength of our study includes the use of validated questionnaires for evaluating resilience, anxiety, depression, and adherence to GFD and data obtained from a multicentre scenario, which may increase the applicability of our results. Our study also has some limitations, which include a lack of data on GFD adherence and symptoms at diagnosis for some patients, the modalities for data collection that were self-reported by patients, and the lack of data on correlation between psychological morbidity and histological recovery of duodenal lesions. In fact, it has been previously shown that patients that have higher levels of anxiety more frequently showed mucosal healing [43].

## 5. Conclusions

Although certainly preliminary and in need of further confirmation by other groups, our findings highlight the relationship between psychological morbidity and resilience and may be helpful to develop specific interventions to strengthen mental health and contrast the psychological burden in a specific subgroup of coeliac patients. Behavioural and cognitive interventions to improve self-confidence, optimism, and resilience could be considered specifically in those coeliac patients with higher anxiety. Interventions to increase resilience may be considered as part of a more holistic approach to coeliac patients in order to decrease the psychological burden of the disease and improve adherence to a GFD and QOL.

## Figures and Tables

**Table 1:** Baseline clinical and sociodemographic features of enrolled patients.

Item	Total, N = 305 (%)
Gender, female	221 (72%)
Age at diagnosis (mean ± SD)	36 ± 16
Age at enrolment (median, IQR)	48 (35–60)
<35 years	68 (22%)
35–55 years	134 (44%)
>55 years	103 (34%)
Clinical pattern at diagnosis [Oslo]	182
Classical	74 (41%)
Non-classical	95 (52%)
Silent	13 (7%)
Disease duration (years, median, IQR)	8 (3–17)
<1 year	34 (11%)
1–5 years	70 (23%)
>5 years	201 (66%)
Educational level	
Academic degree	149 (49%)
No academic degree	156 (51%)
Marital status	
Stable relationship *	196 (64%)
No stable relationship **	109 (36%)
GFD adherence [23]	182
Good (Pavia score ≥3)	162 (89%)
Poor (Pavia score ≤2)	20 (11%)
Clinical response to a GFD	305
Complete	222 (73%)
Partial/absent	83 (27%)
Received instruction on how to follow a GFD?	
Yes	281 (92%)
No	24 (8%)
Membership of Italian Coeliac Association	
Yes	105 (34%)
No	149 (49%)
Previously	51 (17%)
CD-RISC, median (IQR)	69 (41–69)
Low resilience (CD-RISC < 35), N (%)	7 (2%)
High resilience (CD-RISC ≥ 35), N (%)	298 (98%)
CD-QOL, median (IQR)	84 (73–92)
STAI-Y1, median (IQR)	39 (32–50)
STAI-Y2, median (IQR)	40 (33–49)
BDI II, median (IQR)	7 (3–14)

SD: standard deviation; IQR: interquartile range; GFD: gluten-free diet. * This includes 170 married patients, 16 patients who lived together, and 10 engaged patients. ** This includes 93 singles, 14 divorced, 2 others unspecified.

**Table 2:** Relationship between clinical/demographic factors and resilience, anxiety, depression, and quality of life.

	CD-RISC		STAI-Y1		STAI-Y2		BDI II		CD-QOL	
	Median (IQR)	*p*-Value	Median (IQR)	*p*-Value	Median (IQR)	*p*-Value	Median (IQR)	*p*-Value	Median (IQR)	*p*-Value
Gender		0.03		<0.01		0.001		0.001		0.08
M	73 (62–79)		35 (29–45)		36 (31–46)		3 (1–10)		87 (75–93)	
F	68 (58–76)		41 (33–52)		42 (35–50)		8 (3–15)		83 (72–90)	
Clinical pattern at diagnosis		0.68		0.49		0.18		0.04		0.71
Classical	67 (57–76)		41 (30–53)		42 (33–54)		9 (3–17)		83 (71–88)	
Non classical	68 (56–75)		39 (33–49)		42 (35–49)		7 (3–13)		84 (71–90)	
silent	64 (58–73)		33 (31–46)		36 (32–42)		3 (1–5)		85 (73–94)	
Age at enrolment		0.02		0.29		0.01		0.05		0.29
<35 years	67 (53–74)		42 (36–53)		44 (37–56)		9 (4–17)		84 (71–89)	
35–55 years	71 (59–79)		38 (31–49)		39 (32–47)		6 (2–13)		83 (71–92)	
≥55 years	70 (61–76)		39 (32–48)		40 (33–47)		7 (2–13)		85 (76–92)	
Disease duration		0.90		0.41		0.52		0.91		0.71
< 1year	70 (60–77)		45 (36–51)		43 (35–51)		5 (3–14)		85 (70–91)	
1–5 years	67 (59–77)		37 (30–49)		39 (32–47)		8 (2–14)		80 (71–90)	
>5 years	70 (58–76)		39 (32–49)		40 (33–49)		7 (2–13)		85 (74–92)	
Educational level		0.73		0.27		0.76		0.40		0.24
No academic degree	70 (59–77)		39 (31–49)		41 (33–49)		8 (3–15)		85 (76–92)	
Academic degree	68 (58–76)		39 (33–50)		39 (33–49)		7 (2–13)		82 (71–91)	
Marital status		0.03		0.27		0.02		<0.01		0.34
Stable relationship *	70 (60–78)		39 (32–48)		39 (33–47)		6 (2–13)		84 (75–92)	
No stable relationship **	68 (54–75)		40 (33–51)		43 (34–54)		9 (4–16)		84 (70–91)	
Persistent symptoms despite a GFD		0.30		0.12		0.03		<0.01		<0.001
No	69 (59–77)		39 (31–49)		39 (33–48)		7 (2–13)		86 (76–92)	
Yes	69 (54–76)		41 (34–54)		42 (35–55)		8 (4–18)		76 (68–86)	
GFD adherence [23]		0.07		0.11		0.06		0.048		0.19
Poor (Pavia score ≤ 2)	59 (52–70)		48 (36–57)		47 (38–55)		10 (6–22)		77 (68–87)	
Good (Pavia score ≥ 3)	68 (58–76)		39 (32–50)		41 (33–50)		7 (2–14)		84 (72–90)	

GFD: gluten-free diet; SD: standard deviation; * this includes married patients, patients who lived together, and engaged patients. ** This includes singles, divorced, and others unspecified.

**Table 3:** Linear regression analysis and multivariate backward elimination regression analysis showing the relationship between resilience and clinical, psychological, and socio-demographic factors.

Variable	Univariate Regression Analysis Coefficient (95% CI)	*p*-Value *	Multivariate Analysis Coefficient (95%CI) **	*p*-Value *
Gender		0.02		0.59
Male	0 (reference)		0 (reference)	
Female	−4.26 (−7.80, −0.73)		−0.75 (−3.47, 1.98)	
Age at enrolment (years)	0.13 (0.03–0.23)	0.01	0.01 (−0.07, 0.09)	0.79
Clinical pattern at diagnosis				
Classical	0 (reference)	-		
Non-classical	−0.32 (−4.78–4.14)	0.89		
Silent	−3.66 (−12.31–4.99)	0.41		
Disease duration (years)	0.11 (−0.04–0.27)	0.15		
Educational level		1.00		
No academic degree	0 (reference)			
Academic degree	0.00 (−3.19–3.19)			
Marital status		0.02		0.34
Stable relationship	0 (reference)		0 (reference)	
No stable relationship	−4.06 (−7.35, −0.76)		−1.31 (−4.05, 1.41)	
Persistent symptoms despite a GFD	−2.46 (−6.02–1.11)	0.18		
Membership of the Italian Coeliac Association	−1.38 (−4,73–1.97)	0.42		
Good GFD adherence	4.09 (−2.69–10.88)	0.24		
CD-QOL	0.35 (0.23–0.46)	<0.001	0.04 (−0.06, 0.15)	0.40
STAI-Y1	−0.62 (−073,−0.51)	<0.001	−0.88 (−0.25, 0.08)	0.31
STAI-Y2	−0.83 (−0.93, −0.72)	<0.001	−0.98 (−1.14, −0.81)	<0.001
BDI	−0.69 (−0.85, −0.54)	<0.001	0.23 (0.03–0.44)	0.02

GFD: gluten-free diet adherence. * *p*-value <0.05 for significance. ** Backward elimination multivariate regression analysis.

## Data Availability

The original contributions presented in the study are included in the article, further inquiries can be directed to the corresponding author.
